# Spider and Wasp Acylpolyamines: Venom Components and Versatile Pharmacological Leads, Probes, and Insecticidal Agents

**DOI:** 10.3390/toxins16060234

**Published:** 2024-05-21

**Authors:** Gandhi Rádis-Baptista, Katsuhiro Konno

**Affiliations:** 1 Laboratory of Biochemistry and Biotechnology, Institute for Marine Sciences, Federal University of Ceara, Fortaleza 60165-081, Brazil; 2 Institute of Natural Medicine, University of Toyama, Toyama 930-0194, Japan

**Keywords:** biogenic amines, polyamines, acylpolyamines, spider venom, wasp venom, ionotropic glutamate receptors, polyamine transport system, polyamine therapeutics, molecular probes, bioinsecticide

## Abstract

Polyamines (PAs) are polycationic biogenic amines ubiquitously present in all life forms and are involved in molecular signaling and interaction, determining cell fate (e.g., cell proliferation, dif-ferentiation, and apoptosis). The intricate balance in the PAs’ levels in the tissues will determine whether beneficial or detrimental effects will affect homeostasis. It’s crucial to note that endoge-nous polyamines, like spermine and spermidine, play a pivotal role in our understanding of neu-rological disorders as they interact with membrane receptors and ion channels, modulating neuro-transmission. In spiders and wasps, monoamines (histamine, dopamine, serotonin, tryptamine) and polyamines (spermine, spermidine, acyl polyamines) comprise, with peptides and other sub-stances, the low molecular weight fraction of the venom. Acylpolyamines are venom components exclusively from spiders and a species of solitary wasp, which cause inhibition chiefly of iono-tropic glutamate receptors (AMPA, NMDA, and KA iGluRs) and nicotinic acetylcholine receptors (nAChRs). The first venom acylpolyamines ever discovered (argiopines, Joro and Nephila toxins, and philanthotoxins) have provided templates for the design and synthesis of numerous analogs. Thus far, analogs with high potency exert their effect at nanomolar concentrations, with high se-lectivity toward their ionotropic and ligand receptors. These potent and selective acylpolyamine analogs can serve biomedical purposes and pest control management. The structural modification of acylpolyamine with photolabile and fluorescent groups converted these venom toxins into use-ful molecular probes to discriminate iGluRs and nAchRs in cell populations. In various cases, the linear polyamines, like spermine and spermidine, constituting venom acyl polyamine backbones, have served as cargoes to deliver active molecules via a polyamine uptake system on diseased cells for targeted therapy. In this review, we examined examples of biogenic amines that play an essential role in neural homeostasis and cell signaling, contributing to human health and disease outcomes, which can be present in the venom of arachnids and hymenopterans. With an empha-sis on the spider and wasp venom acylpolyamines, we focused on the origin, structure, derivatiza-tion, and biomedical and biotechnological application of these pharmacologically attractive, chemically modular venom components.

## 1. Introduction

**Figure 1:**
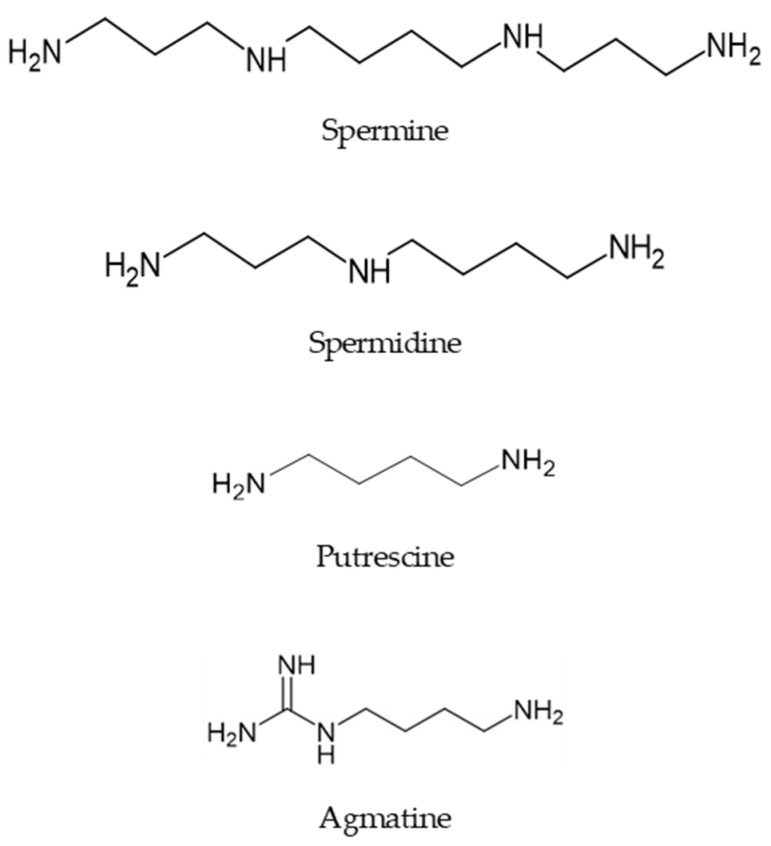
Examples of aliphatic polyamines occurring in organisms of diverse taxa. These PAs are present ubiquitously in nature, from bacteria to humans, including the venom of arachnids and hymenopterans, and regulate numerous biological processes.

Polyamines (PAs) are biogenic polycationic alkylamines ubiquitously found in all living cells and organisms. In animal venoms, polyamines occur chiefly in spiders and certain species of wasps. In eukaryotic cells, the biosynthesis of PAs initiates with the decarboxylation of ornithine and S-adenosyl-methionine; examples include spermine, spermidine, and putrescine [1] (Figure 1). Polyamines play numerous regulatory and functional roles in humans and are critical for human health and diseases. For in-stance, PAs’ biological functions include cell proliferation and differentiation, cell sig-naling and neurotransmission, gene regulation, and apoptosis [2]. An intricate and precise balance in the level of PAs in the cells and bloodstream influences health or disease outcomes, like neuroprotection or neurotoxicity [3,4]. The physiological level of endogenous PAs is associated with controlling chronic disease progression and pro-moting longevity, while high levels, oppositely, are associated with aging and cancer progression [1,2,3,5]. Oscillation in spermine concentration and the ratio between spermine and spermidine are helpful indicators of human health status [6]. Indeed, the polyamine and their metabolites serve as biomarkers for the diagnosis of cancer, stroke, and renal failure [7]. Interestingly, agmatine, a product of L-arginine decar-boxylation, plays an essential role as a regulatory component of the polyamine path-way and is involved in the control mechanism of cell proliferation and the reduction in neoplastic cell expansion [8]. Because the polyamine transport system is upregulated in tumor cells, interrupting polyamine metabolism with antagonists or delivering poly-amine-drug conjugates are interesting pharmaceutical strategies to fight cancer [9]. Additionally, agmatine works as a neurotransmitter in mammals, and experimental evidence indicates its effects on the central nervous system as a neuroprotector in brain injury and damage [10,11]. Since endogenous polyamines also interact with ion channels and neurotransmitter receptors and their regulatory proteins [12,13,14], their altered levels have been implicated in various neurological disorders such as schizo-phrenia, depression, and epilepsy, among other central nervous system (CNS) diseases [4,15,16].

Polyamines are present in the venom of animals, including the venom of various snake species, although their role in the snake venom gland and envenomation is un-known. Despite their presence in snake venom, the quantities of PAs appear insuffi-cient to cause directly harmful systemic effects on the envenomation of human victims [17]. However, it may be possible that aliphatic polyamines in snake venom, especially spermine, could partially contribute to causing hypotension and paralysis on prey by interacting with ionotropic membrane receptors and ion channels [17]. This effect is reasonable to infer since, from numerous studies with distinct biological models, it is understood that polyamine interacting neurotransmitter receptors and -ion channels on cell membranes include ionotropic glutamate receptors (iGluRs: AMPA, NMDA, and kainate), nicotinic and muscarinic acetylcholine receptors (nAchR and mAchR), γ-Aminobutyric acid (GABA) receptors, transient receptor potential cation channels, and inward-rectifier K+-channels [2,12,13,18].

**Figure 2:**
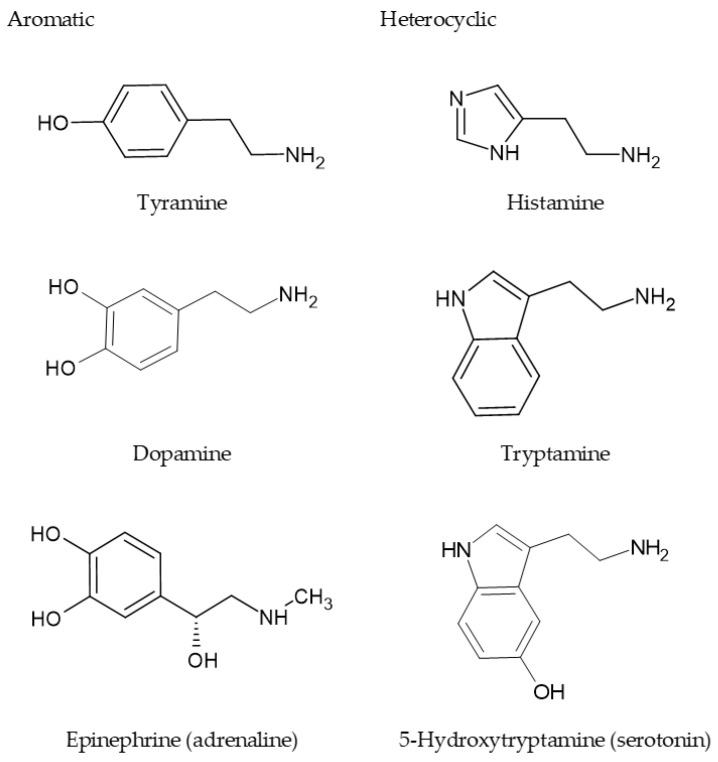
Examples of aromatic and heterocyclic biogenic monoamines. One or more of these bio-genic amines can compose the venom of spiders and wasps.

Polyamines constitute a prominent class of spider (arachnid) and wasp (hyme-nopteran) venom components. Effectively, with hundreds of components, the complex venoms of hymenopterans (bees, wasps, and ants) and arachnids (spiders and scorpi-ons) are a cocktail of substances that, apart from toxic peptides, enzymes, and venom auxiliary proteins, may contain alkaloids, amino acids, biogenic amines, aliphatic and aromatic (acyl) polyamines [19,20,21,22,23,24,25]. Figure 2 shows representative examples of biogen-ic monoamines (aromatic and heterocyclic) that could make up the hymenopteran and arachnid venoms.

## 2. Acylpolyamines in the Venom of Spiders and Wasp

**Figure 3:**
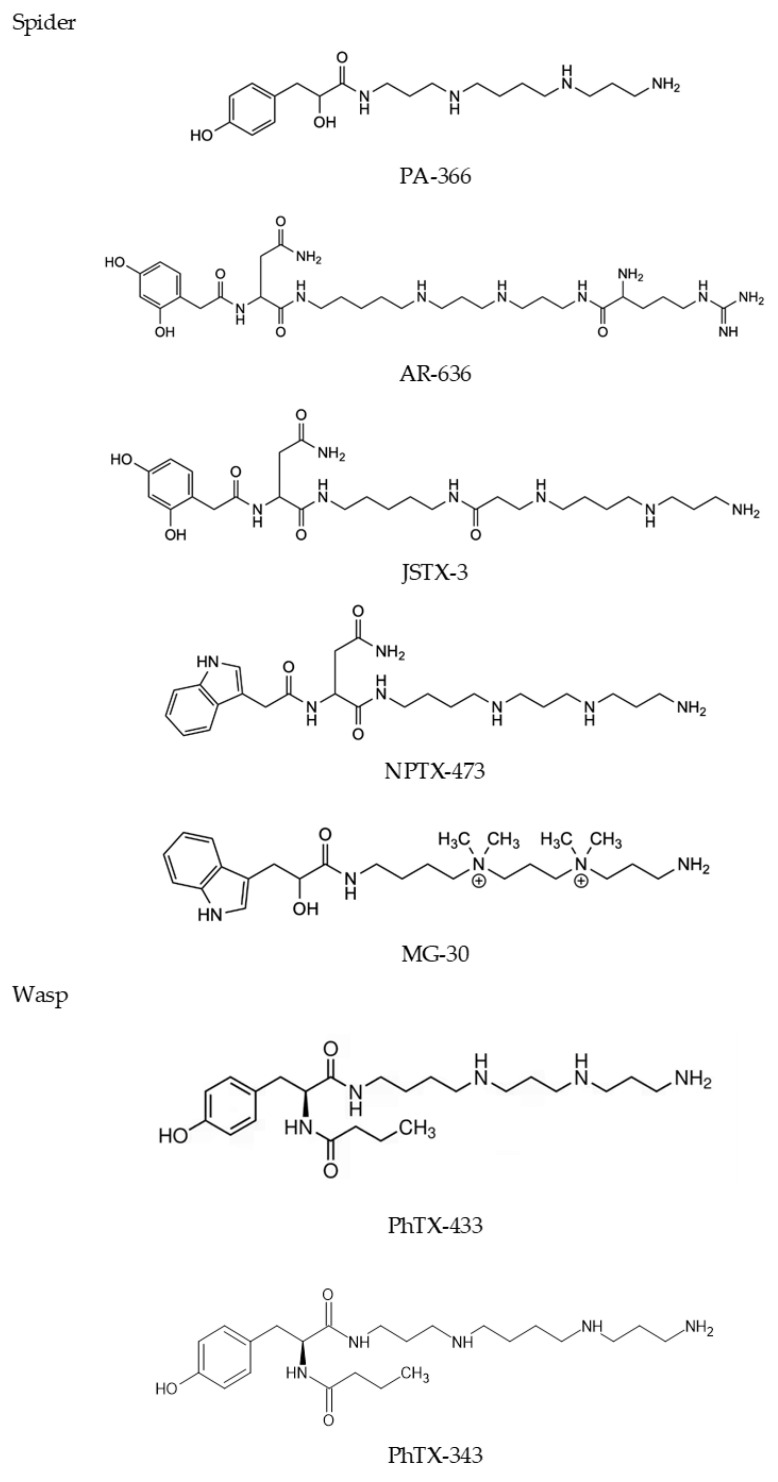
Examples of acyl-polyamines from the spider and wasp venoms. PA-366, 4-OH-PhLac343MG30 from the venom of the tarantula spider *Phlogius* sp. (Theraphosidae); AR-636, 2,4-(OH)₂-PhAcAsn533Arg from the venom of *Argiopa lobata*; JSTX-3, 2,4-(OH)₂-PhAcAsn5ßAla43, from the Joro spider *Nephila clavata* venom; NPTX-473, IndAcAsn433 from a *Nephilengys borbonica* venom gland; and MG-30, IndLac4(Me_2_)3(Me_2_)3^2^⁺ from the spider *Macrothele gigas*. PhTX-433, a butyryltyrosyl-acylpoliamine (philanthotoxin) from the venom of the solitary digger wasp *Philanthus triangulum*. PhTX-343 is a synthetic analogous of native PhTX-433, placed here for comparison. For spider acylpolyamines, the generic nomenclature follows the original names available on the VenoMS database.

Acylpolyamines are venom components exclusively from the venom of spiders and wasps. Acylpolyamines and peptides are the two chief components of spider ven-om, representing two-thirds of the weight of the dried venom, and numerous spider acylpolyamines have been described [26,27,28]. In wasps, acylpolyamines, polyamines, biogenic amines, and peptides compose the venoms’ low molecular weight component fraction [6,29,30]. Chemically and structurally, spider and wasp acylpolyamines con-sist of a hydrophobic aromatic head group (e.g., hydroxy- or dihydroxyphenyl-, or in-dol-3-acetyl- or indol-3-lactyl) in one side of the molecule, linked to the polyamine backbone of variable numbers of methylene groups through a linker (an amino acid, e.g., Asn) or via an amide bond directly, and ending at the other side with primary amines or guanidine (Figure 3). Such structural assemblies of venom acylpolyamines impart to the molecules relatively bulky and hydrophobic head groups and positively charged tails at physiological pH. These latter physical-chemical characteristics have implications for the acylpolyamines’ mechanism of action on ionotropic and ligand re-ceptors on target cells [31].

Usually, the nomenclature of spider and wasp venom acylpolyamines includes let-ters to designate the species of polyamine origin and numerals to indicate the molecu-lar mass (e.g., PA-366 from tarantula species Phlogius sp.) or the number of methylenes between the amino groups of the polyamine moiety (e.g., PhTX-433 from digger wasp). A database of low molecular weight toxins in spider venom, named “VenoMS”, was developed and contained information about their origin, structure, biological activity, and the linked literature. Data regarding spider polyamine and derivatives under mass spectrometry (MS) analysis are also available, with acylpolyamines listed under their generic names [32]. Such generic nomenclature was intended to uniformize the names of polyamine toxins. Thus, the nomenclature designates the acyl polyamine’s head, tail, amino acid linkers, and the methylene units in the polyamine backbones between the amino groups. For instance, the generic name of PA-366 is 4-OH-PhLac343 [32,33].

The molecular targets of most spider and wasp acylpolyamines are glutamatergic excitatory neurons of invertebrate synapses, by which they paralyze insect prey by acting on ionotropic glutamate receptors (iGluRs) [34,35,36,37,38]. The potent inhibition of iGluRs caused by most of the spider and wasp venom acylpolyamines is predominantly voltage-dependent, and the binding occurs in an open-state channel after agonist (glutamate) dissociation [37,39]. Additionally, wasp venom acylpolyamine toxin can target nicotinic acetylcholine receptors (nAChRs), as is the case of phylantoxin-433 (PhTX-433) from the venom of the Egyptian digger wasp, *Philanthus triangulum*, and its structural analogs [23,40,41]. Despite the iGluRs and nAChRs of insects being the primary molecular targets for spider and wasp polyamine toxins, their counterpart receptors in vertebrates are also sensitive to their inhibition, as is the case of mammalian subtypes of iGluRs, i.e., α-amino-3-hydroxy-5-methyl-4-isoxazole propionate (or quisqualate) (AMPA) receptor, N-methyl-D-aspartate (NMDA) receptor, and kainate (KA) receptor, as well as vertebrate muscle- and neuronal type nAChRs [42,43]. An intriguing targeted-selectivity occurs with the acylpolyamine CNS-2130 from the venom of the fishing spider *Dolomedes okefinokensis*, which exceptionally is an antagonist of mammalian L- and R-type voltage-dependent calcium (Ca_v_) ion channel [44].

The fact that glutamate-sensitive ion channels/receptors in excitatory synapsis are the targets for venom acylpolyamines is worthy of note for drug discovery and development. The excessive firing of iGluRs in the human CNS is implicated in several degenerative neurological disorders and brain injuries, like ischemic stroke, Alzheimer’s disease, Parkinson’s disease, amyotrophic lateral sclerosis, ischemia, epilepsy, schizophrenia, depression, and anxiety [45,46]. Thus, venom acylpolyamines and analogs can discriminate and modulate iGluR subtypes, which can be converted into cellular probes and drug leads [47]. Furthermore, spider and wasp venom acylpolyamines targeting nAchRs of insects can be developed for application as selective and potent insecticidal agents, like most commercial insecticides [48,49,50]. The potency and selectivity of venom acylpolyamines toward their targeted receptors are intrinsic characteristics of native acylpolyamines, or they can be adjusted synthetically according to the type of polar head group, the length and type of positively charged polyamine moiety, the N-substituents, and the primary amine or amide terminal [33,51,52,53].

Examples of the first and most studied spider and wasp venom acylpolyamines comprise the group of the argiopines and Joro and Nephila toxins (JSTXs and NPTxs), respectively, originated from the spiders of the genus *Argiopera* and the species *Nephila clavata*. Philanthotoxins comprise the unique and well-studied native venom acylpolyamine of wasp and their analogs (see below). Researchers have synthesized many analogs from their original structures with variable potency and selectivity for their targets, especially nAchRs and iGluRs [54,55,56].

### 2.1. Argiopines

Argiopines are venom 2,4-dihydroxyphenyacetyl-based acylpolyamines isolated from the spider Argiopa lobata that share structural resemblance with argiopinins (and pseudoargiopinins, respectively 4-hydroxy-indol-3-acetyl- and indol-3-acetyl- homo-logs, from the same venom [57]. Notably, argiopine-636, also named argiotoxin-636 (AR636 or ArgTX-636), shares a high structural identity with Joro spider toxin-3 (JSTX-3) and Nephila spider toxin-3 (NPTX-3). Argiopines are potent inhibitors of ionotropic glutamate receptors of, for example, the neuromuscular junctions of invertebrates [57,58], the motoneurones of isolated frog spinal cord [35], and rat cortexes [59]. Argi-opine and pseudoargiopines are naturally N-methylated acylpolyamines. The N-mono-methylated and N-mono-hydroxylated acylpolyamine spider toxins were also identi-fied as venom components of Agelenopsis aperta and Larinioides folium, as they were synthesized using SPS resin and a regioselective reaction [55]. Interestingly, the syn-thesis of N-mono-hydroxylated and N-mono-methylated argiopine and analogs pro-duced highly potent and selective antagonists of NMDA and AMPA iGluRs recombi-nantly expressed in Xenopus laevis oocytes [60], increasing the repertoire of acylpoly-amine structures. In a competitive radioligand assay, ArgTx-636 inhibited alpha-bungarotoxin binding to the muscle-type nAchR of the Pacific electric ray Torpedo cali-fornica, displaying an IC50 comparable with spermine and bis-methylated spermine analog. Notably, ArgTx-636 demonstrates a potent inhibitory activity on neuronal α7 nAChR and human and rat muscle-type α9β10 nAChR [61].

### 2.2. Joro Spider Toxin (JSTX) and Nephila Spider Toxin (NPTX)

Several acylpolyamine toxins were isolated and characterized from the nephilid spiders (Joro spider, Nephila clavate), like Joro spider toxin-3 (JSTX-3) and Nephila pol-yamine toxins-1 and -8 (NPTX-1 and NPTX-8), and the related species, N. maculate, as exemplified by NPTX-3. Nephila toxin-3 (NPTX-3) is an N-(2,4-dihydroxyphenylacety-L-asparaginyl)-N′-(L-arginyl-putreanyl)-cadaverine acylpolyamine that shares struc-tural similarity with JSTX-3, but differs in the head group which comprises an indol-3-acetyl-aromatic instead [62]. Mass spectrometry techniques advanced the characteri-zation of acylpolyamines of Nephila and related spiders directly from crude venom and from a single venom gland [63,64]. The chemical synthesis of analogs of β-alanine-containing polyamines, like JSTX-3 and NSTX-3, and functional analysis lead to the characterization of NPTX-1 and -8 as potent antagonists of kainate receptor (NPTX-1 and -8), and high selectivity to NMDA and AMPA receptors (NPTX-1) [65]. Xiong and colleagues evaluated the structure-activity of dozens of the orb-weaver spider Nephila clavata (Joro spider) polyamine toxins as inhibitors of iGluRs. They found that the other JSTX3, NPTX-1, and NPTX-8 analogs displayed better selectivity and potency for the AMPA receptors than their natural spider polyamine counterparts [39].

### 2.3. Philanthotoxins

Philanthotoxins (PhTXs) are butyryltyrosine derivatives of acylpolyamines and a noncompetitive inhibitor on cation-selective ion channels, including the Ca^2+^-permeable AMPA receptors and the nicotinic acetylcholine receptor (nAChR) from the venom of a solitary wasp species [23,41,66]. A first example of philanthotoxin charac-terized is PhTX-433 purified from the venom gland of the solitary digger wasp Philan-thus triangulum. PhTX-433–a butyryl-tyrosyl-spermine and the synthetic analogs PhTX-334 and PhTX-343 proved to be antagonists of AMPA receptors in insect (locust) leg muscle, being PhTX-334 more potent than the natural polyamine toxin [23]. PhTX-433 and its synthetic analogs, like PhTX-343, are open channel blockers, non-competitive antagonists of iGluRs and nAChRs of insect muscles and CNS, respective-ly, and can inhibit the respective receptors in vertebrate tissues, including in humans [67,68,69,70]. Systematic modification of the PhTx-433’s head group and the polyamine (spermine) tail by design and synthesis yielded analogs with high potency and selectiv-ity for rat AMPA and NMDA iGluRs, as evaluated using patch clamp with recombi-nantly expressed receptors in Xenopus laevis oocytes [71]. Also, analogs of PhTx-343, in which the lipophilic head group was modified with saturated and aromatic rings, dis-played high selectivity and potency toward rat ganglionic nAchRs over brain nAchRs, as demonstrated by electrophysiology measurements with patch-clamped X. oocytes expressing these cloned receptors [72]. Interestingly, despite PhTx-433 being an antag-onist of iGluRs and nAchRs of invertebrates and vertebrates neural systems, it was also reported as an effective inhibitor of E. coli OmpF porin channel and respective electri-cal current [73].

Table 1 lists examples of spider and wasp venom acylpolyamines, their origin, and their targeted membrane receptors.

## 3. Spider and Wasp Acylpolyamines as Versatile Pharmacological Leads, Probes, and Insecticidal Agents

### 3.1. Pharmacological Leads

Polyamines, in general, have received considerable attention from researchers as interesting lead molecules and scaffolds for drug development and delivery for treating chronic and degenerative diseases [9,80,81,82]. Therapeutic strategies can be achieved due to their essential biological roles in modulating cell fates and neurotransmission. For instance, polyamine cell internalization via a specific uptake transport system and interaction with iGlu and nAch receptors allow for the development of polyamine ligands and drug conjugates that control pathophysiological processes.

Because acylpolyamines from spider and wasp venom target essentially nAchRs and iGluRs, using such venom compounds as pharmacological leads may benefit the development of agents for treating pathological conditions that involve glutamatergic synapsis and signaling. Native spider and wasp venom acypolyamines have been molecular templates for designing and preparing numerous analogs with variable selectivity and potency toward their targets.

Linear endogenous polyamines, like spermine, spermidine, and putrescine, ubiquitously found in nature in the cellular and tissue milieu, have generated derivates for biomedical and clinical applications. Regarding the modulation of iGluRs for therapeutic purposes, an example is N^1^-Dansyl-spermine. In vivo, N^1^-Dansyl-spermine is a dose-dependent antagonist of spermine-induced CNS NMDA-mediated excitation in mice, which causes body tremors and tonic convulsions [83]. Such neuroprotective properties qualify this polyamine derivate and other related polyamines for further research on treating neurological disorders like epilepsy [84]. In the same line, parawixin (Pwtx)-1, -2, and -10, 4-hydroxy-indol-3-acetyl-type acylpolyamines of the social orb-web spider *Parawixia bistriata*, have neuroprotective properties, since they, respectively, (1) stimulate L-glutamate uptake through the main transporter in the CNS, (2) inhibit GABA and glycine uptake in synaptosomes, and (3) increase L-glutamate uptake in synaptosomes [85].

The modular polyamine backbone allowed for preparing long linear derivatives with a broad spectrum of antimicrobial activity against multidrug-resistant bacteria [86]. Polyamines with diverse molecular architecture (linear, tripodal, and macrocyclic) and their derivatives with aromatic functional groups, such as 1,3-benzodioxol, ortho- and -para phenol, or 2,3-dihydrobenzofuran, indicated that the topology of the polyamine scaffold is essential for the antimicrobial activity of conjugates [87]. From the hemocytes of the tarantula spider *Acanthoscurria gomesiana*, the bis-acyl polyamine spermidine with antimicrobial and immunomodulatory activity was characterized, and its mechanism of action has been investigated in molecular detail [88,89].

The design, synthesis, and screening of N-substituted and acylspermidine derivates resulted in compounds with anti-proliferative and pro-apoptotic activities on human breast cancer cells and T-lymphoblastic leukemia cells, which could be used to treat solid and blood cancer cells [90]. Spider acylpolyamines have been envisioned as cytotoxic agents, and the structure-activity relationship based on the hydrophobic group translates such functionality. Analogs of the spider (Agel 416, HO-416b) and wasp (PhTx-433) acylpolyamines with modification of the lipophilic head groups and polyamine moiety showed potent antiproliferative activity on MCF-7 and MDA-MB-231 breast cancer cells [91]. A comparison of spider venom acylpolyamines with identical polyamine moieties but with a hydroxyphenyl head group in one acylpolyamine molecule and an indol-based in another can influence the cytotoxic activity in vitro in breast cancer (MCF-7) cell model [92].

Table 2 summarizes examples of using polyamine moiety and venom acylpolyamines as pharmacological leads, probes, potential insecticides, and molecular carriers.

As another exciting example of venom acylpoyamine’s effects on metabolic path-ways and cells and tissues, argiotoxin-636, the potent spider acylpolyamine antagonist of iGluRs, displayed good regulation of melanogenesis by inhibiting the enzymatic ac-tivities of DOPA and 5,6-dihydroxy indole-2-carboxylic acid (DHICA) oxidases [94].

### 3.2. Probes

Apart from modulating ionotropic neurotransmitter receptors and utilizing the linear polyamine moiety as a structural scaffold for drug development, native and syn-thetic analogs of acylpolyamines have been prepared for other biomedical and bio-technological purposes, such as probes for receptor mapping and visualization, carri-ers, and even insecticidal agents.

Fluorescent analogs (e.g., BODIPY-FL-amide conjugates) of the polyamines sperm-ine, spermidine, and putrescine were converted in efficient substrates and probes for testing the mammalian polyamine uptake transport system [103]. Notably, the interac-tion of spider and wasp venom acylpolyamines with their receptors is the rationale be-hind producing probes for target characterization and visualization of responsive live cell populations. In the 1990s, Hashimoto and colleagues synthesized biotinylated PhTX-433 analogs with a higher binding affinity (30–50-fold) than native molecules [104]. Such an analog, bio-C10-PhTX(I2)343-Lys, with a biotin molecule attached to the PhTX-433’s aromatic head group through a C10 spacer and a bifunctional photoaffini-ty probe replacing the terminal lysine, exhibits better performance than the native philanthotoxin relative to the interaction with nAchR. Photolabile analogs of PhTx-343 containing a covalently linked azido group were up to six times more potent antago-nists than native PhTX-343, and were irreversible inhibitors of single locust muscle fi-bers and muscle membranes preparation containing AMPA receptors, when irradiated with U.V. light and stimulated electrically and chemically [95]. Photolabile derivates of phylantoxin prepared with preserved biological activity served for mapping the bind-ing sites in the ligand-receptor interactions, as also exemplified by the PhTx-433 ana-logs 125I-MR44 [41]. Fluorescent probes derived from natural or synthetic compounds are essential resources for investigating biological processes and for application in drug discovery and bioimaging for molecular diagnosis of diseases, among other uses [105]. In this line, two potent glutamate receptor inhibitor analogs of argiotoxin-636, namely ArgTX-75 and ArgTX-48, with an adjusted number of methylene groups in the polyamine backbone, were synthesized with different fluorochromes replacing the polyamine toxins’ head group. The most potent and active argiotoxin analog probe that preserved the inhibitory function of AMPA and NMDA receptors was a 7-amino-4-methylcoumarin derivate. In contrast, the biologically active argiotoxin analog with a linked BODIPY chromophore was used to visualize NMDA receptors in hippocampal live neurons [97]. Similarly, Nishimaru and colleagues prepared fully active fluores-cent-labeled analogs of Madagascar Joro spider toxin (NPTX-594) to use as a probe to visualize glutamate receptors [96]. They replaced the 2,4-dihydroxyphenyacetyl aro-matic head group of NPTX594 for 7-Hydroxycoumarin-4-acetyl fluorophore and Lys residue for N-(4-aminobutyl)glycine to produce a fully active, fluorescent analog that caused paralysis in cricket bioassay. These examples highlight the remarkable use of venom acylpolyamines as probes for mapping and imaging iGluRs. Indeed, dedicated efforts have been made to develop and apply selective ligands for molecular imaging of subtypes of ionotropic glutamate receptors (i.e., NMDA, kainate, and AM-PA/quisqualate) and metabotropic glutamate receptors (mGluRs), aiming to evaluate these receptors in the neurotransmission and pathological processes of neurological disorders [106].

### 3.3. Insecticidal Agents

The de novo designing and synthesis of spiders and wasps acylpolyamines have generated interesting compounds with various structures, potency, selectivity, and us-es. Considering the inhibitory effect on excitatory transmission in insects that cause paralysis, venom acylpolyamines can be employed as native or modified bioinsecti-cides. For instance, analogs designed and synthesized using a philanthotoxin (PhTX-343) and nephilatoxin-8 (NPTX-8) as templates by replacing the tyrosine or asparagine linker for squaryl amino acids proved that hydrophobic phenol moiety of the tyrosin linker of PhTX is critical to cause paralysis. In contrast, analogs of nephilatoxin with glutamine-type squaryl linker (longer chain length linker) showed more potent activity relative to the native spider acylpolyamine toxin in cricket bioassay [98]. Liu et al. compared at the functional level the structures of two dihydroxyphenyl-acylpolyamines from the venom of the spider Araneus ventricosus, namely AVTX-622 and AVTX-623, which differ from each other for only one methylene group in the pol-yamine backbone. They found AVTX-622, lacking one methylene group in the linker region, inhibited voltage-gated sodium channels in neuronal cells of the American cockroach (Periplaneta americana) and displayed a paralyzing potency over ten times stronger than its counterpart, AVTX-623 [51]. The replacement of the tyrosine moiety of PhTX-343 for cyclohexylalanine produced a cyclohexylalanine-PhTX-343 (Cha-PhTX-343) analog that was more potent than the native, unsubstituted venom acylpolyamine in inhibiting, in the nanomolar range, the neuronal signaling through nAchRs [99]. Thus, the potent antagonism of locust nAchR places PhTx-343 analog in the developing processes for obtaining bioactive compounds derived from wasp ven-om. These examples highlight the development and attractive use of spider and wasp venom acylpolyamines as efficacious insecticidal agents for future insect pest man-agement. However, to be converted into advantageous bioinsecticides, venom acylpolyamines and derivates must be highly selective and specific at the pharmaco-logical level and in the context of pest management control.

### 3.4. Carriers

The conjugation of polyamine backbones and various compounds improves the pharmacokinetics of hydrophobic drugs and biologically active groups for cellular de-livery through the polyamine transporter system [102]. In such a case, Ishii and col-leagues developed a polycationic redox-active injectable gel in which polyamines flanked a triblock copolymer to deliver exenatide, a peptide originally from the ven-omous Gila monster (Heloderma suspectum) saliva, to control diabetes [100]. Similarly, to enhance the bioavailability and selectivity of chalcones, which are natural polyphe-nols with multiple biological activities, conjugation with polyamines, like chalcone-N1-spermidine conjugate, was aimed at the cell delivery of these compounds through the polyamine uptake system [101]. In another similar example, based on a polyamine uptake system, the surface of PEG–PLGA nanoparticles modified with spermidine was prepared for the tumor-targeted delivery of the anticancer drug doxorubicin [107]. Given the modularity and structural versatility of aliphatic and acylpolyamines, mul-tifunctional molecules can be designed and synthesized for the targeted delivery of therapeutics via a polyamine uptake system and receptors [108]. Thus, receptor-mediated endocytosis is an interesting cellular entry mechanism for complex polyam-ines, polyamine analogs, and nano polyamines to be further considered in the field [109].

## 4. Conclusions

Biogenic amines, like monoamines (e.g., dopamine, serotonin, and melatonin) and polyamines (spermine, spermidine, and agmatine), are essential in neurotransmission, cell signaling, and neural homeostasis. Beyond neurotransmission, polyamines are multi-functional molecules that regulate numerous biological processes in cells of or-ganisms of different species–from bacteria to plants, and from lower invertebrates to humans. Notably, the levels of endogenous polyamines are implicated in human health or disease status. Spiders and wasps contain (acyl)polyamines in their venoms, which can chiefly modulate nicotinic acetylcholine and ionotropic glutamate receptors. The design and synthesis of venom acyl polyamine analogs with high potency, which selec-tively exert their effect at nanomolar concentrations, can be converted into selective probes to map membrane receptors and channels and to treat neurodegenerative dis-orders. The high potency and selectivity of venom acylpolyamines toward insect neu-ral receptors make these compounds valuable bioinsecticides for pest control. Consid-ering the present examples and the very active research in the field, polyamines and acylpolyamines are interesting compounds for the further investigation and develop-ment of bioactive chemicals for human health and economic benefits.

## 5. Material and Methods

The PubMed search for “polyamines” resulted in 112,514 articles published since 1945. Combinations of terms, like “polyamines and biological function” (11,143), “poly-amines and immunity” (3074), “polyamines and cancer” (17,473), and “polyamines and therapy” (28,756), retrieved thousands of articles primarily published in the last two decades, despite a steady increase in the number of publications since the 1970s.

The search for “polyamines and animal venom” yielded 765 articles published by March 2024. In PubChem (a chemistry database at the National Institutes of Health), “polyamines” resulted in 72 substances, 53 pathways, 2205 bioassays, 5854 patents, and 10,457 articles in the current literature. Searching the combined terms “polyamines and arthropod venom” resulted in 398 articles. The terms “polyamines and spider ven-om” resulted in 194 publications, and “polyamines and wasp venom” resulted in 96. Additionally, the combined terms “acylpolyamines and venom” retrieved 32 articles. Finally, to prepare the manuscript, the inspection and manual selection of articles fol-lowed the electronic engine search, adding relevant references linked to the thematic issue from the current literature.

## Figures and Tables

**Table 1:** Examples of spider and wasp venom acylpolyamines and their target receptors.

Organism	Common Name	Acylpolyamine	Membrane Receptor	Ref.:
Spider				
*Agelenopsis aperta*	Desert grass spider	AG-489	TRPV1 channel **	[74]
*Araneus ventricosus*	Nocturnal orb-weaver spider	AVTX-622	Na_v_ ion channel ^¶^	[51]
*Argiope lobata*	Argiope spider (orb-weaver spider)	ARG-636	iGluR (AMPA) ^¶,^ **; nAChRs **	[31,35,61,75,76]
*Dolomedes okefinokensis*	Fishing spider	CNS-2130	Ca_v_ ion channel ** (L- and R-type)	[44,77]
*Nephila clavata*	Orb-weaver spider (Joro spider)	JSTX-3	iGluR (AMPA) **	[78]
		NPTX-1	iGluR (KA) **	[65]
		NPTX-8	iGluR (KA) **	[65]
*Nephila maculata*	Papua New Guinean orb-web spider	NSXT-3	iGluR ^¶^	[79]
Wasp				
*Philanthus triangulum*	Egyptian digger wasp	PhTX-433	nAChR ^¶^ iGluR (NMDA) ^¶^	[53] [23,69]
		PhTX-343	iGluR (AMPA) ^¶,^ ** iGluR (NMDA) ^¶,^ ** nAChR ^¶,^ **	[40,43,72]

Notes: ^¶^ invertebrate (insects); ** vertebrate/mammalian; Na_v_ ion channel, voltage-dependent sodium ion channel; Ca_v_ ion channel (L- and R-type), voltage-dependent calcium ion channel; iGluRs, ionotropic glutamate receptors: AMPAR, α-amino-3-hydroxy-5-methyl-4-isoxazole propionate (or quisqualate) receptor, NMDA, N-methyl-D-aspartate receptor, and KA, kainate receptor; nAChRs, nicotinic acetylcholine receptor. TRPV1, transient receptor potential cation channel subfamily V (vanilloid). PhTX-343 is a synthetic analogous of the native PhTX-433 from the solitary digger wasp *Philanthus triangulum* venom.

**Table 2:** Examples of spider and wasp venom acylpolyamines and synthetic analogs, including polyamines of distinct sources and their derivates, are useful as phar-macological leads, probes, insecticides, and carriers.

(Acyl-)polyamine Analogs and Derivates	Application	Ref.:
**Pharmacological leads**		
N1-dansyl-spermine	Antagonist of the CNS effects of spermine	[83]
ArgTX-636	Inhibitor of neuronal nAchR and potential analgesic to reduce neuropathic pain	[61]
Parawixin (Pwtx)-1, 2, and -10	Inhibition of seizures and neurodegeneration; neuroprotective and anticonvulsant	[85]
Long linear polyamines derivatives	Antimicrobial agent	[86]
Polyamine-drug conjugates	Antimicrobial agent	[87]
Mygalin (bis-acylpolyamine spermidine)	Antimicrobial and modulator of innate immune responses; anticancer	[88,89,93]
Acylspermidine derivatives	Antiproliferative (anticancer) and pro-apoptotic	[90]
Agel 416, HO-416b and JSTX-3 analogs	Antiproliferative (anticancer) agent	[91]
PA-366 and PA386	Cytotoxic agent for specific lines of cancer cells	[92]
ArgTX-636	Inhibition of melanogenesis	[94]
**Probes**		
Photolabile analogs of PhTx-343	Mapping sensitive receptors	[70,95]
Photolabile analogs of PhTx-433	Mapping ligand-binding sites on receptors	[41]
Fluorescent analogs of NPTX-594	Visualization of acylpolyamine toxin interactions with iGluRs	[96]
Fluorescent analogs of ArgTX-636	Imaging of iGlu receptors in neurons	[97]
**Insecticides**		
Glu-type squaryl-NPTX derivatives	Paralysis on insects, glutamatergic signaling disruptor	[98]
AVTX-636	Paralysis on insects, inhibition of Nav ion channels	[51]
Cyclohexylalanine-PhTX-343	Paralysis on insects, inhibition of locust nAchR	[99]
**Carriers**		
Polyamine polyion complexes	Delivery of antidiabetic peptide	[100]
Chalcone-polyamine conjugates	Anticancer therapy acting via the upregulated polyamine transport system	[101]
Polyamine-drug conjugates	Delivery of bioactive payloads through the polyamine transporter system	[102]

Notes: Aliphatic polyamines (e.g., spermine and spermidine) and venom acylpolyamines were used as lead compounds for structural modification and application. Mygalin is not a component of the tarantula *Acanthoscurria gomesiana* spider, but is from the hemocytes.

## Data Availability

The original contributions presented in the study are included in the article, further inquiries can be directed to the corresponding authors.
